# Dolutegravir quantification in wistar rat tissues following chronic administration^☆^

**DOI:** 10.1016/j.heliyon.2023.e22541

**Published:** 2023-11-18

**Authors:** N. Henning, C. Smith, T.A. Kellermann

**Affiliations:** Division of Clinical Pharmacology, Department of Medicine, Faculty of Medicine and Health Sciences, Stellenbosch University, Cape Town, South Africa

**Keywords:** Dolutegravir, Rat tissues, LC-MS/MS, Dose adjustment, Adverse effects

## Abstract

Dolutegravir (DTG) has been introduced into first line combination antiretroviral therapy (ART) for HIV/AIDS. Penetration of ART into HIV reservoirs is essential to prevent continuous replication of HIV. However, accumulation of DTG in HIV reservoirs could be contributing to the adverse effects reported. We have developed and applied liquid chromatography tandem mass spectrometry (LC-MS/MS) methods to quantify DTG in wistar rat biological matrices following chronic DTG administration**.** DTG was detected using a Shimadzu 8040 triple quadrupole-mass spectrometer. The methods developed were in the concentration ranges of 17.5–8000 ng/mL for plasma and 15.5–16 680 ng/g for tissue matrices. Mean plasma DTG concentrations in the current study closely corresponded to plasma DTG levels reported in humans after chronic treatment. Plasma and tissue DTG concentrations were generally higher in females compared to male wistar rats, but these differences were nullified after correcting for body and organ size. Plasma DTG levels correlated with tissue DTG concentrations in the liver and gastrocnemius muscle tissue. Data suggest that body size – rather than sex – may be a major risk factor determining adverse outcomes of patients on the current DTG dosing strategy which does not account for differences in body mass. Furthermore, plasma DTG was not correlated with adipose tissue DTG concentration. This suggests that adipose may be a primary site for longer term inflammatory dysregulation and adverse outcome following DTG treatment.

## 1 Introduction

Dolutegravir (DTG) - an integrase strand inhibitor (INSTI) – is currently used in first line treatment for the management of human immunodeficiency virus (HIV) infection [1,2]. The United States Food and Drug Administration (FDA) has approved DTG for treatment of HIV in a broad patient population [1,3]. DTG, which is commonly administered as a compounded drug – together with tenofovir and lamivudine [4] – was originally reported to be very well tolerated. However, more recently, it has been associated with increasing reports of adverse effects [5]. Several side effects – most with an inflammatory character – have been reported for DTG-containing ART regimens, especially in female patients. The tissues that seem to be affected with highest prevalence are the gastrointestinal tract and the brain [[6], [7], [8]].

For an ART drug to be effective, it should be able to reach its target without causing adverse effects. Penetration of reservoirs by ART is essential to prevent the continuous replication of the virus [9]. Plasma vs tissue levels of DTG may indicate the ability of the ART to penetrate HIV tissue reservoirs and thus prevent the continuous replication of the virus. A complete picture can only be gained from assessment of various biological matrices for penetration of ART into these potential viral reservoirs, which includes the gut, adipose and the brain [10]. In terms of risk profile, the distribution or potential accumulation profile of DTG at multiple tissue sites could provide information on tissues most at risk for side effects in the longer term. Interestingly, INSTI have been linked to higher risk for immune reconstitution inflammatory syndrome [11]. In addition, DTG in particular has been linked to increased pro-inflammatory signalling by pre-adipocytes [12] as well as activation of neutrophils [13]. Importantly, these interactions were observed at non-cytotoxic concentrations attainable within a therapeutic setting by using a 50 mg DTG dose [13]. This could increase the risk of adverse and/or chronic complications, should DTG accumulate in tissue. Given these facts, as well as the known high-risk profile of most other ART in terms of co-morbidity and longer-term chronic disease risk, it is a priority to elucidate the exact nature of DTG distribution and its potential differential accumulation in tissue compartments. Since DTG is not administered as monotherapy, it is difficult to assign its relative contribution to these side-effects without assessing its effects in isolation. Given the relative inability of low-to middle-income countries (LMIC) to carry the additional cost of management of chronic side-effects, research in this context is a priority.

When analysing the effect of DTG on different tissues, it is important to consider the distribution of the drug. The proven ability of DTG to penetrate tissues could contribute to its capacity for targeting persistent low-rate replication of HIV in its tissue reservoirs [6]. However, improved penetration into reservoirs and off-target tissues may predispose patients to co-morbidity risks in particular tissue compartments. In the early years of use, DTG was reported to have a low incidence of adverse effects, being well tolerated and exhibiting infrequent drug-drug interactions [14]. More recent reports have suggested that adverse effects related to DTG are more frequent than previously reported [5]. This may be due (at least in part) to wider application of the drug, or more frequent prescription of DTG to patients already taking other chronic medications, but currently insufficient data preclude firm conclusions on the reasons for this. Despite the causes, given the high-risk profile of most other ART in terms of co-morbidity and longer-term chronic disease risk, it is a priority to elucidate the exact nature of DTG distribution and its potential differential accumulation in tissue compartments, as this may inform on risk profile. For example, the majority of side effects reported for DTG involve the neurological and/or gastrointestinal systems [5,6,14]. Thus, the ability to quantify levels of DTG in these tissues may provide insights into whether these tissues are most vulnerable to side-effects due to relatively low penetration of DTG into these known viral reservoirs (i.e. relatively larger viral effect), or due to high penetration of DTG, which may suggest undesired off-target effects of DTG itself. We hypothesised that plasma and tissue distribution of DTG may provide insight into potential sex- or tissue-specific risk for adverse outcome in patients.

Here we describe the development and application of a novel method to quantify DTG concentrations in wistar rat plasma, adipose, brain, muscle and liver tissue by using reverse phase liquid chromatography coupled with mass spectrometry. The methods develop were used to quantify DTG in different rat matrices to determine distribution and potential sex- or tissue-specific relative accumulation of DTG following a 12-week DTG administration.

## 2 Materials and methods

### 2.1 Ethical considerations

All animal experiments were conducted according to the guidelines and ethical standards set out by the South African National Standard (SANS: 10 386:2008) for the care and use of animals for scientific purposes 10 386:2008 and the Research Ethics Committee: Animal Care and Use (REC: ACU) of Stellenbosch University (ref# ACU-2021-22 035). All experiments were conducted at Stellenbosch University.

### 2.2 Rodent study

Wistar rats were housed in the Stellenbosch University Animal Facility under normal husbandry conditions. Starting at seven weeks old, the 12 female and 12 male rats (average body mass 185 ± 26.6 g) were weight-matched into four groups; a female control group (who received placebo jelly cubes), a female intervention group (who received DTG infused jelly cubes), a male control group (who received placebo jelly cubes) and a male intervention group (who received DTG infused jelly cubes). Rats were allowed to acclimatise to cage grouping, handling and experimental procedures for one week before initiation of the DTG intervention.

#### 2.2.1 Administration of dolutegravir

The DTG used for the intervention study was extracted from (50 mg) Olegra® dolutegravir sodium tablets (Aurobindo Pharma, South Africa). A human equivalent dose (HED) for rats was calculated according to previous methods [15,16] with some adaptations to the formula:

$$HED\left(mg/kg\right)=Animal\;dose\left(mg/kg\right)\times \frac{AnimalK_{m}}{HumanK_{m}}$$

The $K_{m}$ factor is determined by dividing the body mass (kg) by BSA (m^2^). The equation was adapted by adjusting the average human body mass from 60 kg to 70 kg [17] and the average human BSA from 1.60 m^2^ to 1.73 m^2^ [18]. In addition, the average rat mass was adjusted to the average body mass of 5 month-old wistar rats (0.219 kg) and average BSA of 0.036 m^2^ [19]. Using the revised calculation, a HED dose of 4.7 mg/kg was determined. The intervention groups were therefore administered 1 mg DTG orally, once daily (instead of dose continuously adjusted for body mass) to more closely mimic the standard practise of administration of a 50 mg daily DTG dose in human patients weighing more than 35 kg [20]. DTG administration was continued for a period of 12 weeks, with the final dose administered 24 h prior to endpoint sample collection.

Chronic DTG administration was achieved by daily jelly cube administration for a period of 12 weeks, starting at the age of 8 weeks. Jelly cubes were prepared daily by mixing 810 mg unflavoured gelatine with 16 g of Moir's raspberry jelly powder in 30 mL boiling water. Once the jelly mixture was dissolved it was transferred to a 1 cm^3^ silicon mould. The placebo blocks were prepared by using drug-free jelly. The intervention blocks were prepared by aliquoting 400 μL jelly into the mould, followed by the 1 mg extracted DTG and then another 400 μL jelly to ensure the DTG was covered entirely. The jelly cubes were left to set at 4 °C for at least 2 h. One entire jelly block was hand fed directly to each rat to ensure that the full dose was consumed. The animals were accustomed to placebo jelly blocks for one week before intervention initiation.

#### 2.2.2 Tissue collection and processing

General wellness was assessed daily and body mass biweekly. One week prior to experimental endpoint, blood glucose was measured by tail-vein needle prick and a Contour® Plus Blood Glucose Monitoring System (Bayer, Germany). At the end of the 12-week intervention, rats were killed by intraperitoneal injection of sodium pentobarbital overdose (200 mg/kg). Aortic puncture was executed and blood anticoagulated in EDTA Vacutainer ® (BD-Plymouth, UK) tubes. EDTA whole blood was centrifuged at 21 °C for 10 min at 1000×*g* and subsequently the plasma layer aliquoted and frozen at - 20 °C until analysis.

The left hemisphere of the brain, the left retroperitoneal adipose depot, the tip of the left lateral lobe of the liver, and the left gastrocnemius muscle were excised and immediately snap frozen in liquid nitrogen. The tissue samples were then stored at −80 °C until analysis. Samples were only collected at study endpoint.

### 2.3 Analysis of DTG accumulation

#### 2.3.1 Reagents

DTG (D528800) and its isotope-labelled internal standard (IS), DTG-d4 (D528802), were purchased from Toronto Research Chemicals (Toronto, ON, Canada). LC-MS grade acetonitrile (ACN) and methanol (MeOH) were purchased from ROMIL Ltd. (Cambridge, UK) and formic acid (FA) from Fischer Chemicals (New Hampshire, USA). Ultrapure water was produced from a Synergy® water purification system (Merck KGaA, Darmstadt, Germany). Dimethyl sulfoxide (DMSO) was purchased from Sigma-Aldrich (Missouri, USA).

#### 2.3.2 LC-MS/MS analysis

Analysis was conducted on a SHIMADZU 8040 triple quadrupole-mass spectrometer (SHIMADZU, Kyoto, Japan) connected to a SHIMADZU Prominence LC system. The system consisted of a LC-20ADXR solvent delivery system, Nexera XR SIL-20AXR autosampler and CTO-20A column oven. The analytes were chromatographically resolved on an Agilent Poroshell 120 EC-C18 (3.0 × 100 mm, 2.7 μm) column. Acquisition was set in positive electrospray ionization mode using multiple reaction monitoring. The following transitions for protonated products [M+H]^+^ were monitored: *m/z* DTG, 419.95 → 277.05; *m/z* DTG-d4, 423.95 → 279.00. Argon was the collision-induced dissociation gas, delivered at 230 kPa. The electrospray ionization parameters are as follows; nebulizing gas flow (3 L/min), desolvation line temperature (250 °C), heating block temperature (400 °C), interface voltage was 4.50 kV and drying gas flow (15 L/min).

For plasma analysis, chromatographic separation was carried out with a 3 min isocratic mobile phase consisting of water containing 0.1 % FA (mobile phase A) and ACN containing 0.1 % FA (mobile phase B) (40:60, v/v) at a flow rate of 0.4 mL/min. The column temperature was set at 30 °C and 2 μL of extracted plasma was injected. The autosampler temperature was set to 10 °C. The needle rinse consisted of ACN containing 1 % DMSO. DTG and DTG-d4 eluted at 1.80 and 1.79 min, respectively. For tissue sample analysis, DTG was chromatographically separated using a gradient elution profile with the same mobile phases used for the isocratic method at a flow rate of 0.4 mL/min. The gradient started at 55 % mobile phase B and increased to 100 % mobile phase B over 3 min, held at 100 % mobile phase B until 4 min and reduced to 55 % mobile phase B until 4.50 min. The system was re-equilibrated at 55 % mobile phase B for a total run time of 7.50 min. The column temperature was set at 30 °C and 5 μL of extracted adipose, muscle and liver sample were injected onto the column. For brain sample analysis, 2 μL was injected. The autosampler temperature was set at 10 °C. The needle rinse was ACN with 1 % DMSO with a rinse dip time of 10 s. During the gradient analysis of adipose, muscle and liver, DTG and DTG-d4 eluted at 1.98 and 1.97 min, respectively. From the extracted brain tissue, DTG and DTG-d4 eluted at 2.02 and 2.01 min, respectively.

#### 2.3.3 Preparation of calibrators, quality controls and internal standard

Stock solutions (SS) of DTG were prepared in ACN at a concentration of 2 mg/mL. DTG-d4 IS SS were prepared in MeOH at 1 mg/mL. SS were aliquoted and stored at −80 °C. The 2 mg/mL DTG SS was serially diluted with ACN to prepare the final working solutions (WS) of calibration standards. Similarly, quality control (QC) WS were prepared by diluting a 2 mg/mL SS with 100 % ACN. All WS were stored at −80 °C. Calibration standards and QCs were prepared on the day of extraction from frozen WS aliquots. On the day of analysis, an eight-point plasma calibration curve, six-point brain calibration curve and nine-point adipose, muscle and liver calibration curve were prepared by spiking pooled blank matrix with WS not exceeding 5 % (v/v) of the total volume. Blank (analyte free) matrix - plasma, brain-, adipose-, muscle- and liver tissue - were collected from wistar rats not being treated with DTG. EDTA whole blood was collected from several rats and was centrifuged at 21 °C for 10 min at 1000×*g*, subsequently the plasma layers were removed and pooled for calibration standard and QC preparations. Each organ/tissue were mixed 1:5 (w/v) with ACN:H_2_O (70:30, v/v) containing 0.2 % FA and homogenized with a tissue homogenizer (4 cycles, 6.95 m/s, 15 s interval with 1 min dwell time between each cycle, Bead Ruptor Elite, OMNI International, Georgia, USA) and the homogenate pooled for use in matrix matched calibration standard and QC preparations. The plasma calibration concentrations ranged from 17.5 to 8000 ng/mL, the adipose, muscle and liver concentrations ranged from 3.05 to 3336 ng/mL and the brain calibration curve ranged from 42.1 to 3336 ng/mL. Peak area ratio (analyte peak area/IS peak area) versus nominal concentration of analyte were plotted to generate a calibration curve using a quadratic regression with a 1/C^2^ weighting. The plasma QC samples were prepared at 6400 ng/mL (QC H), 3200 ng/mL (QC M) and 50 ng/mL (QC L). The adipose, muscle and liver QC samples were prepared at 2669 ng/mL (QC H), 1345 ng/mL (QC M) and 9.00 ng/mL (QC L). The brain QC samples were prepared at 2669 ng/mL (QC H), 1345 ng/mL (QC M) 60 ng/mL (QC L). The QC samples were prepared to verify the calibration curves. Calibration standards and QCs were prepared in duplicate for each analytical run when analysing samples and had to have a percentage accuracy of 85–115 and percentage coefficient of variation <15 %. Method robustness evaluation results are published elsewhere [21].

#### 2.3.4 Sample pre-treatment

Plasma samples were thawed on ice. A volume of 190 μL drug-free plasma was spiked with 10 μL of the respective WS to generate calibration standards and QCs. For the protein precipitation, 200 μL cold ACN containing DTG-IS at 62.5 ng/mL was added to 50 μL plasma calibration standard, QC or test sample. The samples were vortexed for 2 min, followed by centrifugation for 10 min at room temperature at 16 000×*g*. The supernatant was removed and centrifuged again for 10 min at room temperature at 16 000×*g.* The resulting supernatant was transferred to a 96-well plate and 2 μL was injected for analysis.

Brain, adipose, muscle and liver tissue were removed from −80 °C, weighed, mixed 1:5 (w/v) with ACN:H_2_O (70:30, v/v) containing 0.2 % FA and homogenized with a tissue homogenizer (4 cycles, 6.95 m/s, 15 s interval with 1 min dwell time between each cycle, Bead Ruptor Elite, OMNI International, Georgia, USA). A volume of 190 μL tissue homogenate was individually spiked with 10 μL working stock for calibration standard and QC preparation. A volume of 700 μL cold ACN containing 32 ng/mL DTG-IS was added to the 200 μL calibration standards, QC and test samples. The samples were vortexed for 2 min, followed by centrifugation for 10 min at room temperature at 16 000×*g*. Thereafter, the samples were eluted through Oasis PRiME HLB sorbent wt. 60 mg 3 cc cartridges (Waters™ Massachusetts, USA). The cartridges were conditioned with 1 mL ACN prior to sample loading. A volume of 700 μL of the sample was loaded onto the cartridges followed by elution twice with 500 μL MeOH containing 0.1 % FA. The eluent was evaporated to dryness under a stream of nitrogen at 35 °C and reconstituted in 350 μL ACN. The samples were transferred to 96 well plates and 5 μL of the adipose, muscle and liver samples was injected for analysis while 2 μL of the brain sample was injected.

### 2.4 Statistical analysis

Data was obtained from instrument software (LabSolutions version 5.109) and analysed using Microsoft excel version 16.54 and GraphPad Prism 9.4.1 software. Data is presented as the mean ± the standard deviation (SD). Outliers were determined using ROUT (Q = 1 %). Distribution of data was assessed using Shapiro-Wilk normality test. Statistical analyses of end-point measurements included 2-way ANOVA with Tukey's multiple comparison test. DTG concentration in the plasma and tissues detected between males and females was analysed using an unpaired *t*-test. Linear relationships were determined using Pearson's correlation or Spearman's correlation (r) depending on distribution of data.

## 3 Results

**Fig. 1:**
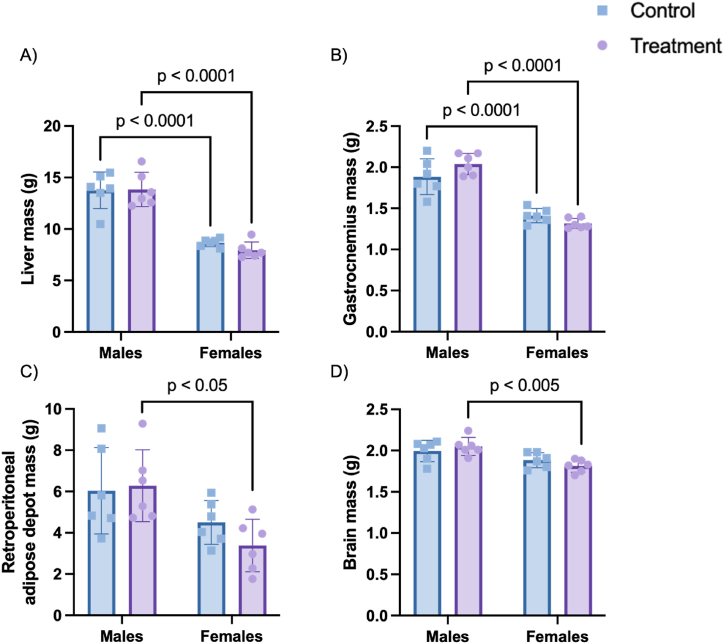
Mass of (A) liver, (B) left gastrocnemius muscle, (C) left retroperitoneal adipose depot and (D) brain from male and female wistar rats after 12-week DTG administration. Data is expressed as mean ± SD, n = 6. Statistical analysis: 2-way ANOVA with Tukey's multiple comparison test.

Male rats exhibited significantly higher body mass (p < 0.0001) than females at the end of the protocol, with no apparent effect of DTG (Supplementary Fig. 1). Fasting blood glucose was similar and within the normal range for fasting blood glucose of wistar rats (3.95 ± 1.31 mmol/L) [22] for all experimental groups (Supplementary Fig. 2). In line with the larger body mass in males, organ masses were also larger in males, but with no significant effect of DTG (Fig. 1).

**Fig. 2:**
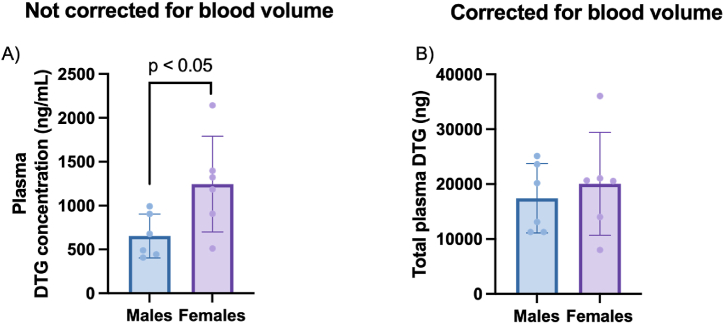
Average DTG concentration detected in plasma following a 12-week DTG intervention. (A) Not corrected for blood volume. (B) Corrected for blood volume. Data are expressed as mean ± SD, n = 6. Statistical analysis: unpaired *t*-test.

The LC-MS/MS methods developed were used to quantify DTG in plasma and accumulation of DTG in various tissue compartments at the study endpoint. In terms of DTG distribution, DTG-administered female rats exhibited significantly higher plasma DTG concentration vs males, that was nullified after adjustment for calculated total blood volume (Fig. 2). Total blood volume was calculated based on a healthy rat having 64 mL of blood per kg of bodyweight [23].

**Fig. 3:**
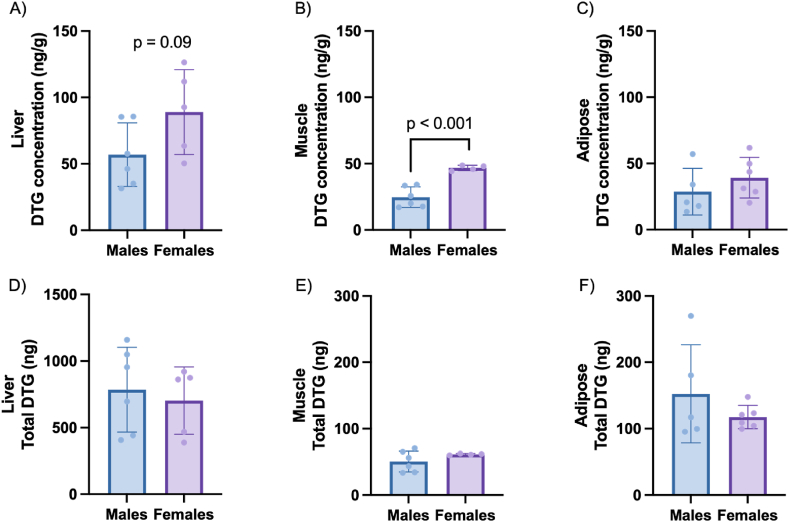
DTG concentration detected in (A) tip of left lateral lobe of liver (B) left gastrocnemius muscle and (C) retroperitoneal adipose depot tissue following a 12-week DTG administration. Total amount of DTG detected corrected for organ mass in the (D) tip of the left lateral lobe of the liver, (E) left gastrocnemius muscle and (F) retroperitoneal adipose depot of rats following a 12-week DTG intervention size. Data are expressed as mean ± SD. Statistical analysis: unpaired *t*-test.

The highest tissue concentration of DTG was detected in the liver (Fig. 3A). Concentrations in adipose and muscle tissue were in a similar range (Fig. 3 B, C), but approximately 50 % lower than those in liver, while no DTG was detected in the brain. In addition, in all tissues with detectable concentrations of DTG, average concentrations were higher in female than male rats, although this difference only reached statistical significance for muscle tissue, where data were less variable. Similar to findings in plasma, correction of tissue DTG concentrations for total organ mass, also nullified this difference (Fig. 3 D, E, F). Individual concentrations of DTG detected in each compartment for the different rats are summarised in Supplementary Table 1.

**Fig. 4:**
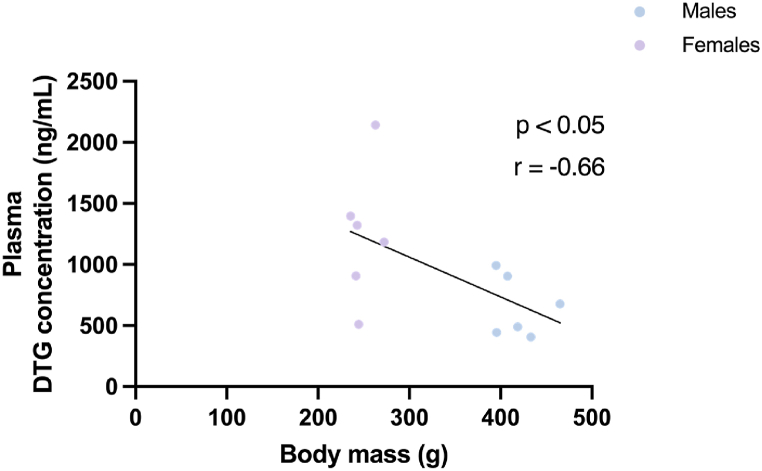
The correlation between DTG plasma concentration (ng/ml) and body mass (g) of 20-week-old wistar rats. Statistical analysis: Spearman's correlation.

Correlation analyses shed further light on the relationship between body/organ mass and DTG levels measured. Given the relatively low statistical power for correlations in a sex-specific manner, we opted not to perform such correlations. However, correlation data is presented in a manner distinguishing between males (indicated in blue on graphs) and females (in purple). When considering both sex groups together, lower body mass is correlated with higher plasma concentrations of DTG (Fig. 4). However, qualitative assessment of the sex groups independently of each other on the same graph, suggest that at least in the female group, there is no correlation between body mass and plasma DTG concentration.

**Fig. 5:**
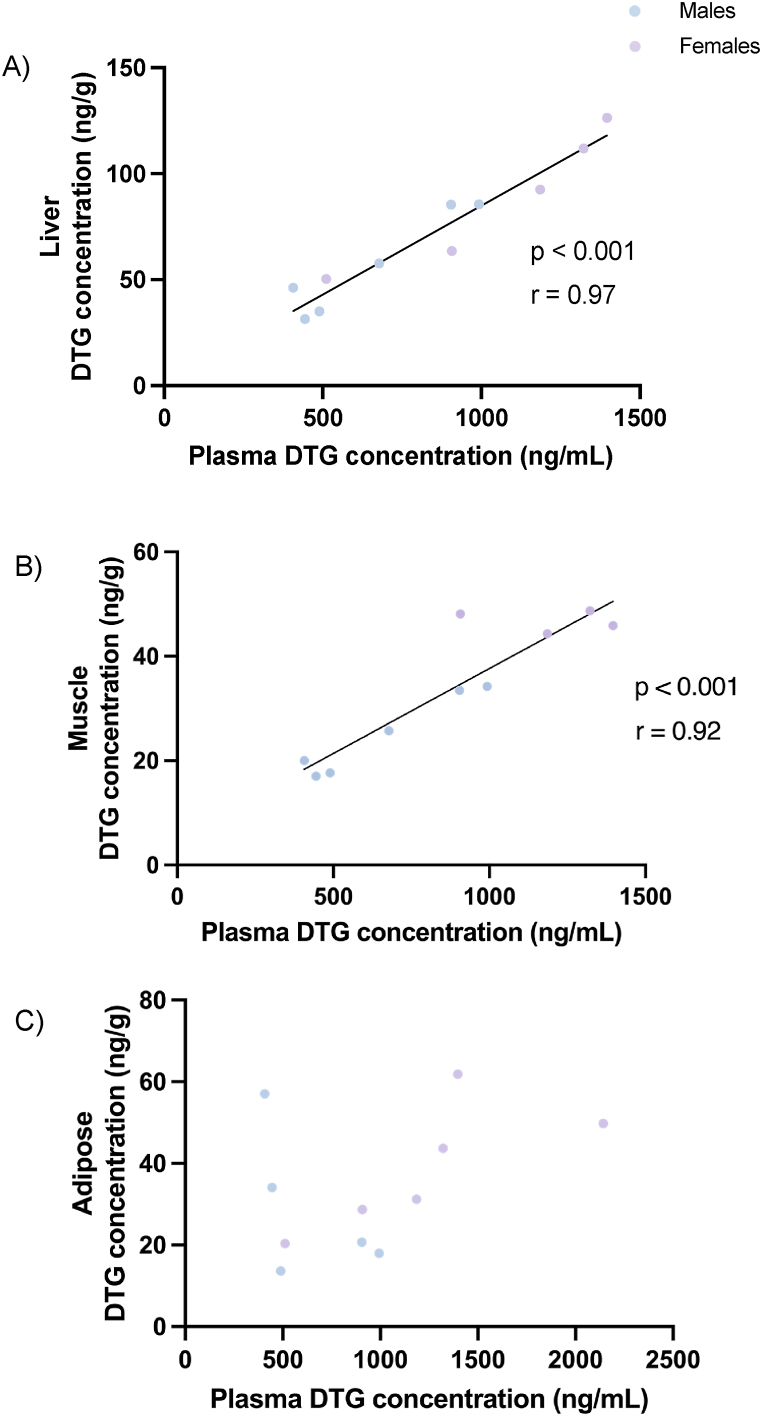
Correlation between DTG concentration (ng/g) in the (A) tip of the left lateral lobe of the liver, (B) left gastrocnemius muscle and (C) left retroperitoneal adipose depot vs plasma concentration (ng/mL) following a 12-week DTG intervention in rats.

When considering tissue DTG concentrations however, clear positive correlations existed between plasma DTG and both liver and muscle, but not adipose (Fig. 5 A, B, C). These data suggest that plasma DTG concentration cannot serve as proxy for events at the level of adipose tissue but may correspond closely to changes in DTG in some other body compartments. Of further interest, in muscle and liver tissue the correlation patterns seem similar for males and females. However, in adipose tissue, while a similar positive correlation as that seen in muscle and liver seems evident in females, no such correlation pattern was observed in males.

## 4 Discussion

The current study describes LC-MS/MS methods for the quantification of DTG in wistar plasma and several tissues. In addition, we report plasma and tissue concentrations of DTG after chronic administration in male and female rats, illustrating plasma DTG concentrations closely corresponding to DTG levels reported in humans [24]. This suggests that an accurate, physiologically relevant dosing strategy was employed in the rodent study, which validates interpretations made from this pre-clinical model.

Current dosing practise in the clinical environment is a single dose administered daily to all patients with a body mass equal to or higher than 35 kg [20]. At first glance, the significantly higher plasma and tissue levels of DTG observed in female rats in the current study seem to suggest a sex difference in DTG pharmacodynamics. However, when the plasma and tissue concentrations were corrected for total blood volume and total organ mass respectively, these differences were nullified, suggesting that body size, rather than sex, may be the major determining factor at play. Indeed, correlation analysis indicated a significant, inverse correlation between plasma DTG and body mass. The fact that male rats weighed significantly more than female rats (approximately 60 % more in the current study), achieved a larger effect size than what is likely possible to achieve in human cohorts, demonstrating the benefit of pre-clinical studies in pharmacovigilance research. Furthermore, this model allowed for elucidation of similar trends at tissue level. Thus, although current study design did not allow for a volume of distribution calculation, the consistent trend of higher DTG in smaller body size may suggest that individuals of smaller stature may be exposed to relatively greater DTG concentrations both in circulation and at tissue level, when administered the same dose as individuals with relatively larger body size, irrespective of sex. This may lead to increased adverse effects in patients with smaller body size.

In support of this notion, women generally have a smaller blood volume than men [25], and current world data supports the assumption that in general, men weigh more and are taller than women in the same population [26]. This interpretation of current data is in line with a number of more recent studies reporting that women and older individuals (i.e. those with relatively smaller stature) seem to experience higher incidences of side effects and discontinuation of DTG treatment [8,27,28]. This was seldom reported in initial and/or earlier clinical trials including DTG, but may have gone unnoticed due to the fact that women frequently comprised less than 25 % of study participants in earlier studies [[29], [30], [31]]. The fact that weight and body mass index are often reported as averages of the study cohort and not by sex, excludes many studies from contributing to wider evaluation of this possibility. Nevertheless, current data suggest that dosing strategy of DTG should be revisited to account for smaller body size.

Interestingly, the statistic on body size differences between males and females is true for all regions across the world, except in Southern Africa, where - although men are still significantly taller than females - average body mass of females generally exceeds that of men [26]. This difference can largely be explained by relatively larger subcutaneous adipose stores in these females. The fact that DTG levels in adipose tissue seemed to deviate from other compartments in terms of its relationship to body size in the current study, may have specific significance in this population, although a number of factors may have contributed to the outcome.

Firstly, pathological expansion of adipose tissue can lead to excessive lipid accumulation and in turn an immune/inflammatory response [13], which has been shown to be exacerbated by DTG [12]. Females are considered to have greater subcutaneous adiposity than males, while males tend to accumulate abdominal, visceral adipose tissue [32]. The current study suggests that DTG may accumulate in adipose tissue relative to other tissues, possibly leading to a risk of adipose tissue physiological dysregulation as result. However, no subcutaneous adipose depots were analysed in this current study, and this would be required before a definite interpretation can be made on the relevance of the result in visceral tissue.

Another consideration is the fact that DTG is highly protein bound [33]. In plasma, DTG is 99 % bound to plasma proteins, mainly albumin and to a lesser extent alpha-1-acid-glycoprotein (AGP) [34]. Both albumin and AGP are also produced in adipose tissue [35,36], as well as other proteins that make up the extracellular matrix (ECM) such as proteoglycans, fibrous tissues, as well as regulatory proteins such as adipocyte fatty acid binding protein, C-reactive protein, adiponectin and leptin [37]. Dysfunction in the expression of these proteins and the ECM have been linked to increased adiposity and metabolic syndrome [37,38] - two conditions that have been associated with DTG treatment [29,[37], [38], [39]]. Thus, the lack of correlation between DTG levels in adipose vs plasma, may suggest that the 12-week administration of DTG had dysregulated the adipose protein profile, and thus DTG binding in this tissue. This would suggest that adipose tissue could be more vulnerable to an inflammatory outcome in response to DTG. Although human females are known to have a higher adipose protein content than males [38,40] male rodents accumulate significantly more adipose depots than females of a similar age [41]. Thus, although no sex-differences in adipose DTG levels were observed in the current study, a rodent model may not be the ideal model for adipose-related studies on sex differences in this context. Proteomics analysis of adipose protein profile in humans treated with DTG may shed more light on this possibility.

In terms of tissue penetration, current data confirms good tissue penetration of DTG, with the exception of brain tissue. DTG is mainly metabolized in the liver by hepatic glucuronidation by UDP-glucuronosyltransferase [14]. It was therefore expected that more DTG would be detected in the primary site of elimination, as was indeed the case. This result is similar to recent findings on rodent liver DTG concentrations following administration of a similar dose [42].

The fact that adipose tissue exhibited a significantly higher DTG content than muscle tissue suggests that high tissue adipose content is not a limiting factor for DTG penetration into brain tissue. Reports on DTG penetration into brain tissue are variable. Relatively low levels of DTG (5 ng/g tissue) have been reported in murine brain tissue previously [43] in conjunction with plasma DTG levels very similar to those in the current study. This could suggest that the method employed for detection in the current study was not sufficiently sensitive to detect these low levels, as the limit of detection was ∼200 ng/g for brain tissue homogenate. However, in contrast, another study in male wistar rats administered 45 mg/kg (in nano formulation) DTG via intra-muscular injection and no DTG was detected in the brain, using an LC-MS method with a 25 ng/g limit of detection [42]. Collectively, despite potential limitations in detection methods, these studies indicate that DTG is unlikely to readily penetrate the blood-brain barrier in the absence of viral infection. However, the effects of HIV-infection on blood brain barrier permeability to DTG remains to be investigated.

Finally, current data suggests that in the absence of HIV infection and other ART, DTG does not seem to contribute to weight gain or dysregulation of glucose. These data correspond to the more recent literature in which it is proposed that DTG alone is not responsible for weight gain; evidence has been provided suggesting that tenofovir alafenamide (TAF) potentially enhances weight gain. It has been suggested that TAF and DTG may have an additive effect on weight gain, but this should be further investigated [44].

## 5 Conclusions

A sensitive and robust LC-MS/MS method has been developed for the accurate quantification of DTG in rat plasma and tissues at physiologically relevant levels. Current data presented suggest that DTG on its own does not contribute to weight gain or glucose dysregulation. However, data also suggest a specific vulnerability of adipose tissue to DTG, which warrants further elucidation. Importantly, data suggest that body size may be a major risk factor determining adverse effect outcome, with a potential added sex effect in the context of adipose tissue sensitivity to DTG. We recommend that dosing strategy be revised to allow administration of DTG in a manner more closely adjusted for body mass.

## Funding sources

This work was supported by The 10.13039/501100004513Harry Crossley Foundation and the South African 10.13039/501100001321National Research Foundation (grants to 10.13039/100014300NH and 10.13039/100013015CS respectively).

## CRediT authorship contribution statement

**N. Henning:** Data curation, Formal analysis, Investigation, Methodology, Project administration, Software, Validation, Visualization, Writing – original draft, Writing – review & editing. **C. Smith:** Conceptualization, Data curation, Funding acquisition, Investigation, Project administration, Resources, Supervision, Visualization, Writing – original draft, Writing – review & editing. **T.A. Kellermann:** Conceptualization, Data curation, Formal analysis, Funding acquisition, Investigation, Methodology, Project administration, Resources, Software, Supervision, Validation, Visualization, Writing – original draft, Writing – review & editing.

## Declaration of competing interest

The authors declare the following financial interests/personal relationships which may be considered as potential competing interests.
