# Supplementary material for: Dolutegravir quantification in wistar rat tissues following chronic administration
Source: Heliyon. 2023 Nov 18;9(12):e22541. doi: 10.1016/j.heliyon.2023.e22541 (PMC10709360; doi:10.1016/j.heliyon.2023.e22541)
Supplement: Multimedia component 1 [file mmc1.docx]

**Dolutegravir quantification in wistar rat tissues following chronic administration**

N. Henning, C. Smith, T.A. Kellermann*

Division of Clinical Pharmacology, Department of Medicine, Faculty of Medicine and Health Sciences, Stellenbosch University, Cape Town, South Africa

***Supplementary tables and figures***

*Supplementary table 1: Individual DTG concentrations detected in plasma and tissue compartments.*

*Supplementary figure 1: Change in body mass of wistar rats over 12-week DTG administration protocol.*

*Supplementary figure 2: Fasting blood glucose (mmol/L) in the control and 12-week DTG administered groups.*

*Supplementary table 1: Individual DTG concentrations detected in plasma and tissue compartments.*

| *Sex* | *Rat* | *Plasma DTG concentration (ng/mL)* | *Liver DTG concentration (ng/g)* | *Muscle DTG concentration (ng/g)* | *Adipose DTG concentration (ng/g)* |
| --- | --- | --- | --- | --- | --- |
| *Female* | *1* | 2 143 | **346* | **100* | 49.8 |
|  | *2* | 1 185 | 92.6 | 44.3 | 31.2 |
|  | *3* | 512 | 50.3 | **319* | 20.3 |
|  | *4* | 907 | 63.5 | 48.1 | 28.7 |
|  | *5* | 1 322 | 112 | 48.7 | 43.7 |
|  | *6* | 1 396 | 126 | 45.9 | 61.8 |
| *Male* | *1* | 905 | 85.4 | 33.5 | 20.7 |
|  | *2* | 407 | 46.2 | 20.0 | 57.0 |
|  | *3* | 678 | 57.6 | 25.7 | **298* |
|  | *4* | 993 | 85.6 | 34.2 | 18.0 |
|  | *5* | 445 | 31.4 | 17.0 | 34.1 |
|  | *6* | 490 | 35.1 | 17.7 | 15.5 |

**sample not included in analysis due to classification as outlier (ROUT Q= 1%)*


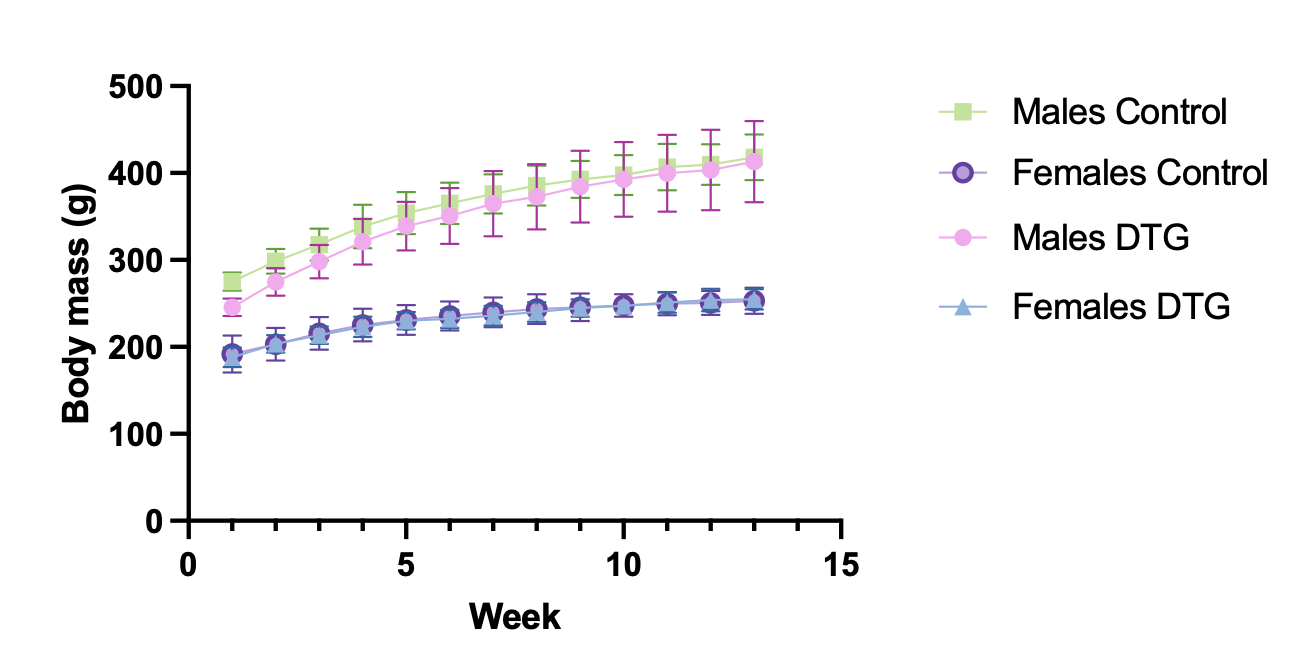


*Supplementary figure 1: Change in body mass of wistar rats over 12-week DTG administration protocol. Data is expressed as mean ± SD. n=6 per group. Statistical analysis: 2-way ANOVA with Tukey’s multiple comparison test.*

*
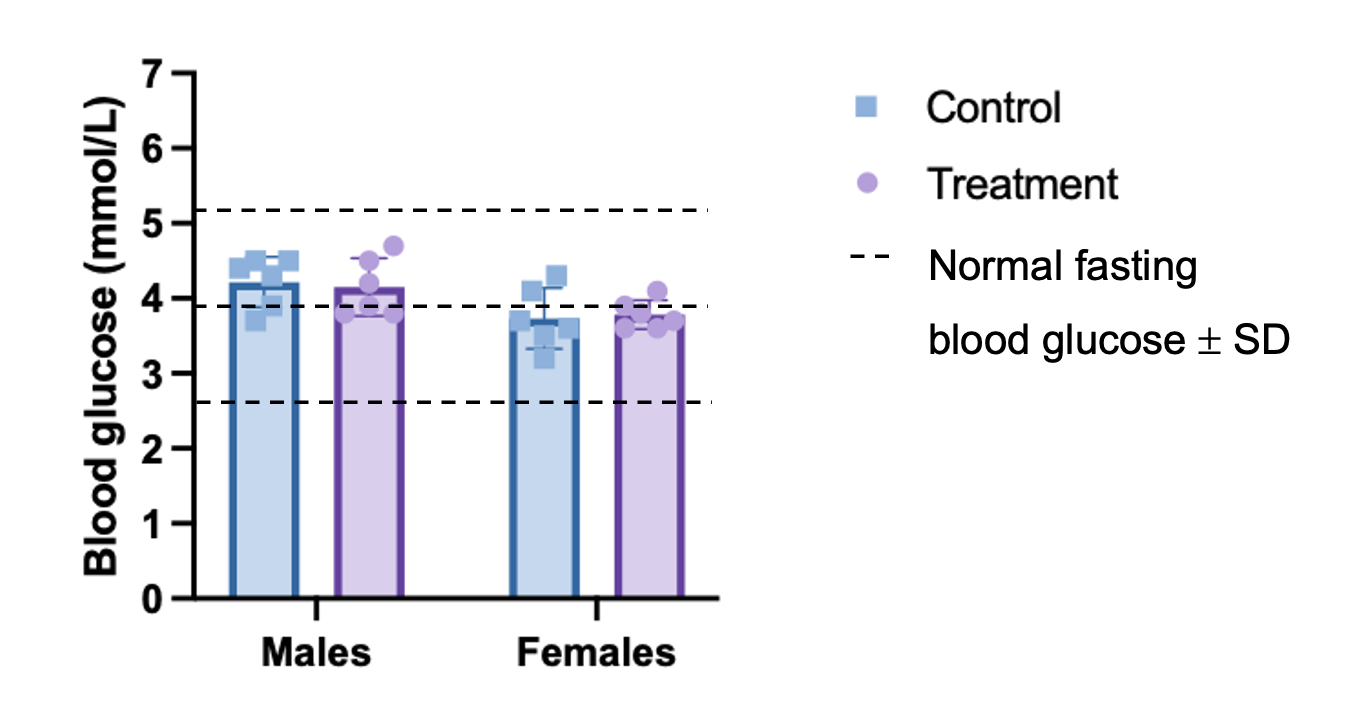
*

*Supplementary figure 2: Fasting blood glucose (mmol/L) in the control and 12-week DTG administered groups. Data is expressed as mean ± SD, n=6 per group. Statistical analysis: 2-way ANOVA with Tukey’s multiple comparison test. Dotted lines represents the normal range for fasting blood glucose in rats 3.95 ± 1.31 mmol/L* (Wang et al., 2010)*.*
